# Diethyl 6,13-dioxo-5,7,12,13b,13c,14-hexa­hydro-6*H*,13*H*-5a,6a,12a,13a-tetra­azabenz[5,6]azuleno[2,1,8-*ija*]benz[*f*]azulene-13b,13c-dicarboxyl­ate 1,2-dichloro­ethane solvate

**DOI:** 10.1107/S1600536809041373

**Published:** 2009-10-17

**Authors:** Hong-Xia Liu, Zhi-Guo Wang

**Affiliations:** a School of Environmental Science and Engineering, Huangshi Institute of Technology, Huangshi 435003, People’s Republic of China; b School of Chemical and Materials Engineering, Huangshi Institute of Technology, Huangshi 435003, People’s Republic of China

## Abstract

In the title inclusion compound, C_26_H_26_N_4_O_6_·C_2_H_4_Cl_2_, the solvent mol­ecule occupies a cavity inside the clip-type mol­ecule which is based on the glycoluril skeleton with two ethyl acetate substituents on the convex face of the glycoluril system. The dihedral angle between the aromatic rings of the host is 43.59 (4)° and the centroid–centroid distance is 6.741 (5) Å. The 1,2-dichloro­etane mol­ecule adopts a *gauche* conformation enabling it to participate in C—H⋯π inter­actions with the host. The packing motif in the title compound differs from that observed in the crystal structures of the host and in the benzene solvate. The host mol­ecules are linked into tapes by π–π stacking inter­actions (centroid–centroid distance = 3.733  Å) and are further assembled into layers *via* C—H⋯O inter­actions. One of the ethyl groups is disorded over two positions with site-occupancy factors of 0.702 (14) and 0.298 (14).

## Related literature

For the related structures, see: Chen *et al.* (2007 ▶); Hof *et al.*,(2002 ▶); Hu *et al.* (2007 ▶); Isaacs & Fettinger (1999 ▶); Wang *et al.* (2006 ▶). 
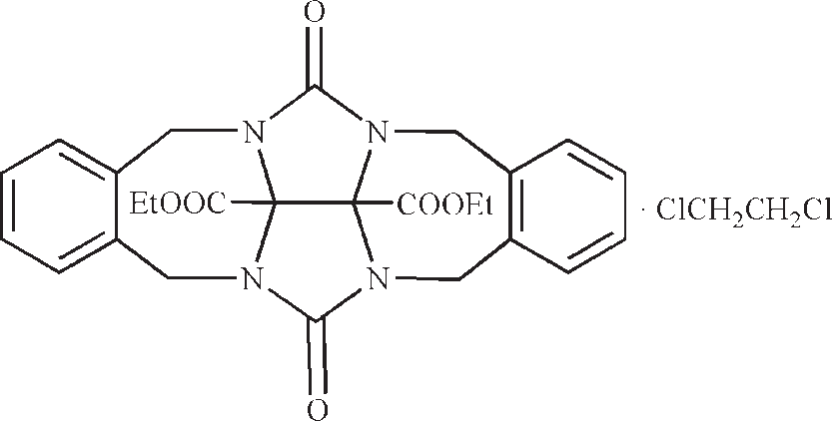


## Experimental

### 

#### Crystal data

C_26_H_26_N_4_O_6_·C_2_H_4_Cl_2_
*M* *_r_* = 589.46
Triclinic, 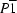

*a* = 8.9468 (10) Å
*b* = 11.1544 (13) Å
*c* = 15.6260 (18) Å
α = 69.257 (2)°
β = 82.688 (2)°
γ = 76.892 (2)°
*V* = 1418.5 (3) Å^3^
*Z* = 2
Mo *K*α radiation
μ = 0.28 mm^−1^
*T* = 292 K
0.30 × 0.20 × 0.10 mm

#### Data collection

Bruker SMART CCD area-detector diffractometer
Absorption correction: none
10171 measured reflections
4947 independent reflections
4056 reflections with *I* > 2σ(*I*)
*R*_int_ = 0.020

#### Refinement

*R*[*F* ^2^ > 2σ(*F* ^2^)] = 0.063
*wR*(*F* ^2^) = 0.188
*S* = 1.04
4947 reflections
383 parameters
28 restraints
H-atom parameters constrained
Δρ_max_ = 0.69 e Å^−3^
Δρ_min_ = −0.64 e Å^−3^

### 

Data collection: *SMART* (Bruker, 2001 ▶); cell refinement: *SAINT* (Bruker, 2001 ▶); data reduction: *SAINT*; program(s) used to solve structure: *SHELXS97* (Sheldrick, 2008 ▶); program(s) used to refine structure: *SHELXL97* (Sheldrick, 2008 ▶); molecular graphics: *SHELXTL* (Sheldrick, 2008 ▶); software used to prepare material for publication: *SHELXTL*.

## Supplementary Material

Crystal structure: contains datablocks I, global. DOI: 10.1107/S1600536809041373/gk2232sup1.cif

Structure factors: contains datablocks I. DOI: 10.1107/S1600536809041373/gk2232Isup2.hkl

Additional supplementary materials:  crystallographic information; 3D view; checkCIF report

## Figures and Tables

**Table 1:** Hydrogen-bond geometry (Å, °)

*D*—H⋯*A*	*D*—H	H⋯*A*	*D*⋯*A*	*D*—H⋯*A*
C28—H28*B*⋯*Cg*2	0.97	2.55	3.476 (3)	160
C5—H5⋯O5^i^	0.93	2.59	3.393 (4)	145
C7—H7*B*⋯O5^i^	0.97	2.50	3.359 (3)	148
C14—H14*A*⋯O2^ii^	0.96	2.57	3.514 (9)	168
C14′—H14*F*⋯O2^ii^	0.96	2.47	3.351 (6)	153

Symmetry codes: (i) 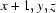; (ii) 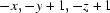. *Cg*2 is the centroid of the C21–C26 ring.

**Fig. 1.**
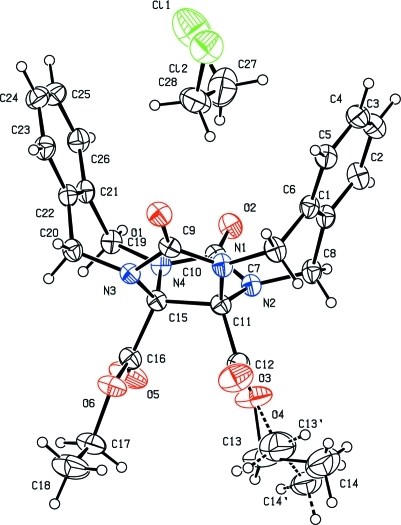
The molecular structure of the title compound with 50% probability displacement ellipsoids.

**Fig. 2.**
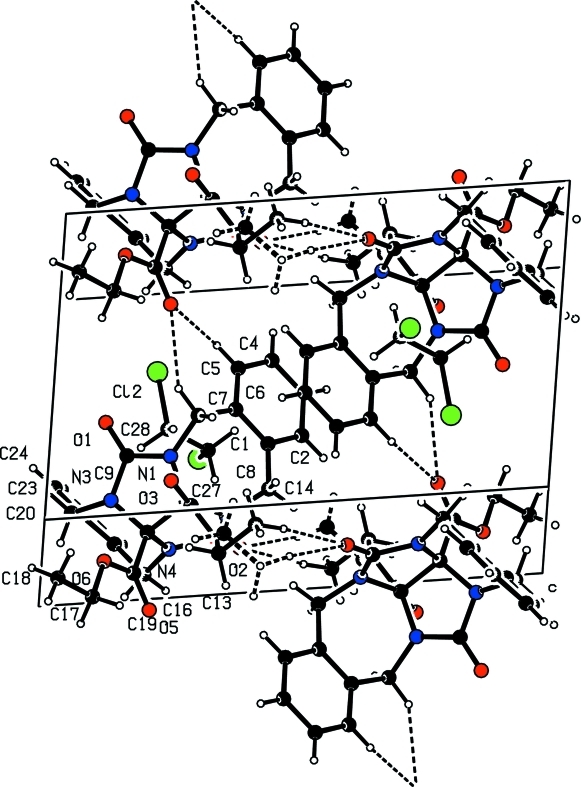
Crystal packing of the title compound

## supplementary crystallographic information

### Comment

Molecular clips based on the glycoluril skeleton, which have a well defined geometry due to the rigidity of the fused rings, have been prepared for a wide variety of supramolecular applications, including molecular recognition (Hu *et al.*, 2007), molecular assemblies (Hof *et al.*, 2002), crystal engineering (Wang *et al.*, 2006; Chen *et al.*, 2007),

The title host compound is a kind of molecular receptor, possessing a well defined U-shaped cavity formed by the glycoluril framework with two aromatic side walls. The crystal structures of monoclinic apohost obtained from dichloroethane solution (C2/c, Z' = 1/2; Wang *et al.*, 2006) and monoclinic benzene solvate, C_26_H_26_N_4_O_6_.0.25C_6_H_6_, (P2_1_/c, *Z' *= 2; Isaacs *et al.*, 1999) have been already reported. Here, we report the structure of a new solvate (P-1, Z' = 1), C_26_H_26_N_4_O_6_.C_2_H_4_Cl_2_, crystallized from 1,2-dichoroethane/methanol (3:1 vol.) solution.

In the triclinic pseudopolymorph, the asymmetric unit contains one host molecule and one solvent molecule of 1,2-dichloroethane (Fig.1). There is a C-H···π interaction betweeen the host and the guest. The distance between atom H28B and *Cg*2 (the centroid of the C21—C26 ring) is 2.67 Å (Table 1).

As solvent molecule occupies the cavity, the title compound exhibits different packing motif than the previously known two monoclinic forms. In Isaacs structure, the molecule was kined into three-dimensional network structure by π-π stacking, C—H···π and C—H···O hydrogen-bonds. In our previously reported structure of the apohost, the ethyl group occupied the cavity with C—H···π  interactions and the molecules were linked into layers to the *ab* plane by four pairs of C—H···O hydrogen-bonds parallel .

The title molecules are linked into tapes by π-π stacking interactions. The benzene rings *Cg*1 (C1—C6) in the molecules at (*x*, *y*, *z*) and (1 - *x*, 1 - *y*, 1 - *z*) are strictly parallel, with an interplanar spacing of 3.589 Å, a ring centroid separation of 3.733 Å and a centroid offset of 1.027 Å. In addition, intermolecular C—H···O (Table 1) hydrogen-bonds link molecules into two-dimensional layer structure.

### Experimental

The host compound was synthesized as reported previously (Wang *et al.*, 2006). Crystals of the title compound were obtained by slow evaporation at 293 K of 1,2-dichoroethane/methanol (*vol.* 3:1) solution .

### Refinement

The H atoms from methyl groups were placed in calculated positions, with C—H=0.96 Å, and refined to fit the electron density [*U*_iso_(H)=1.5*U*_eq_(C)]. Other H atoms were placed in calculated positions, with C—H = 0.93Å (aromatic) and 0.97Å (methylene), and refined in riding mode [*U*_iso_(H)=1.2*U*_eq_(C)]. One of the ester ethyl groups (C13,C14) is disordered over two positions. Restraints were imposed on the geometry of the disordered ethyl group and anisotropic displacement parameters. The occupanicies of the disordered atoms C13/C13' and C14/C14' refined at 0.702 (14)/0.298 (14).

### Crystal data

**Table:**

C_26_H_26_N_4_O_6_·C_2_H_4_Cl_2_	*Z* = 2
*M**_r_* = 589.46	*F*(000) = 616
Triclinic, *P*1	*D*_x_ = 1.380 Mg m^−^^3^
Hall symbol: -P 1	Mo *K*α radiation, λ = 0.71073 Å
*a* = 8.9468 (10) Å	Cell parameters from 5260 reflections
*b* = 11.1544 (13) Å	θ = 2.3–26.1°
*c* = 15.6260 (18) Å	µ = 0.28 mm^−^^1^
α = 69.257 (2)°	*T* = 292 K
β = 82.688 (2)°	Block, colorless
γ = 76.892 (2)°	0.30 × 0.20 × 0.10 mm
*V* = 1418.5 (3) Å^3^	

### Data collection

**Table:**

Bruker SMART CCD area-detector diffractometer	4056 reflections with *I* > 2σ(*I*)
Radiation source: fine-focus sealed tube	*R*_int_ = 0.020
graphite	θ_max_ = 25.0°, θ_min_ = 2.0°
φ and ω scans	*h* = −10→10
10171 measured reflections	*k* = −13→13
4947 independent reflections	*l* = −18→18

### Refinement

**Table:**

Refinement on *F*^2^	Primary atom site location: structure-invariant direct methods
Least-squares matrix: full	Secondary atom site location: difference Fourier map
*R*[*F*^2^ > 2σ(*F*^2^)] = 0.063	Hydrogen site location: inferred from neighbouring sites
*wR*(*F*^2^) = 0.188	H-atom parameters constrained
*S* = 1.04	*w* = 1/[σ^2^(*F*_o_^2^) + (0.1032*P*)^2^ + 0.8923*P*] where *P* = (*F*_o_^2^ + 2*F*_c_^2^)/3
4947 reflections	(Δ/σ)_max_ = 0.001
383 parameters	Δρ_max_ = 0.69 e Å^−^^3^
28 restraints	Δρ_min_ = −0.64 e Å^−^^3^

### Special details

**Table:**

Geometry. All e.s.d.'s (except the e.s.d. in the dihedral angle between two l.s. planes) are estimated using the full covariance matrix. The cell e.s.d.'s are taken into account individually in the estimation of e.s.d.'s in distances, angles and torsion angles; correlations between e.s.d.'s in cell parameters are only used when they are defined by crystal symmetry. An approximate (isotropic) treatment of cell e.s.d.'s is used for estimating e.s.d.'s involving l.s. planes.
Refinement. Refinement of *F*^2^ against ALL reflections. The weighted *R*-factor *wR* and goodness of fit *S* are based on *F*^2^, conventional *R*-factors *R* are based on *F*, with *F* set to zero for negative *F*^2^. The threshold expression of *F*^2^ > σ(*F*^2^) is used only for calculating *R*-factors(gt) *etc*. and is not relevant to the choice of reflections for refinement. *R*-factors based on *F*^2^ are statistically about twice as large as those based on *F*, and *R*- factors based on ALL data will be even larger.

### Fractional atomic coordinates and isotropic or equivalent isotropic displacement parameters (Å^2^)

**Table:**

	*x*	*y*	*z*	*U*_iso_*/*U*_eq_	Occ. (<1)
C1	0.3563 (3)	0.4517 (3)	0.43467 (17)	0.0419 (6)	
C2	0.4101 (3)	0.3329 (3)	0.5006 (2)	0.0527 (7)	
H2	0.3424	0.2944	0.5472	0.063*	
C3	0.5627 (4)	0.2702 (3)	0.4988 (2)	0.0585 (8)	
H3	0.5962	0.1900	0.5434	0.070*	
C4	0.6644 (3)	0.3268 (3)	0.4308 (2)	0.0558 (8)	
H4	0.7670	0.2856	0.4294	0.067*	
C5	0.6127 (3)	0.4452 (3)	0.3650 (2)	0.0489 (7)	
H5	0.6816	0.4833	0.3191	0.059*	
C6	0.4600 (3)	0.5090 (3)	0.36552 (18)	0.0418 (6)	
C7	0.4116 (3)	0.6394 (3)	0.29185 (19)	0.0477 (7)	
H7A	0.3833	0.7071	0.3197	0.057*	
H7B	0.4982	0.6589	0.2487	0.057*	
C8	0.1895 (3)	0.5177 (3)	0.44266 (18)	0.0453 (6)	
H8A	0.1394	0.4605	0.4949	0.054*	
H8B	0.1866	0.5974	0.4552	0.054*	
C9	0.2946 (3)	0.6387 (2)	0.15541 (18)	0.0398 (6)	
C10	0.0566 (3)	0.4531 (2)	0.34105 (17)	0.0372 (6)	
C11	0.1265 (3)	0.6567 (2)	0.27998 (17)	0.0378 (6)	
C12	0.0736 (3)	0.7930 (3)	0.2899 (2)	0.0508 (7)	
C14	−0.0459 (15)	0.9472 (8)	0.4212 (6)	0.128 (4)	0.702 (14)
H14A	−0.0458	0.8751	0.4776	0.192*	0.702 (14)
H14B	−0.0985	1.0265	0.4314	0.192*	0.702 (14)
H14C	0.0581	0.9543	0.3994	0.192*	0.702 (14)
C13	−0.1260 (11)	0.9249 (6)	0.3514 (6)	0.095 (3)	0.702 (14)
H13A	−0.2332	0.9230	0.3705	0.114*	0.702 (14)
H13B	−0.1203	0.9933	0.2925	0.114*	0.702 (14)
C15	0.0392 (3)	0.6362 (2)	0.20652 (16)	0.0368 (5)	
C16	−0.1181 (3)	0.7301 (3)	0.18476 (19)	0.0444 (6)	
C17	−0.2468 (4)	0.9420 (3)	0.1062 (3)	0.0680 (9)	
H17A	−0.3236	0.9100	0.0863	0.082*	
H17B	−0.2870	0.9616	0.1614	0.082*	
C18	−0.2110 (6)	1.0594 (4)	0.0345 (3)	0.0979 (15)	
H18A	−0.1399	1.0935	0.0562	0.147*	
H18B	−0.3036	1.1239	0.0187	0.147*	
H18C	−0.1658	1.0379	−0.0187	0.147*	
C19	−0.0402 (3)	0.4324 (3)	0.20547 (18)	0.0429 (6)	
H19A	−0.1262	0.4892	0.1699	0.052*	
H19B	−0.0778	0.3591	0.2512	0.052*	
C20	0.1259 (3)	0.6119 (3)	0.05267 (17)	0.0428 (6)	
H20A	0.1919	0.6513	0.0009	0.051*	
H20B	0.0205	0.6467	0.0350	0.051*	
C21	0.0802 (3)	0.3813 (3)	0.14224 (17)	0.0419 (6)	
C22	0.1590 (3)	0.4658 (3)	0.07154 (17)	0.0406 (6)	
C23	0.2683 (3)	0.4133 (3)	0.01628 (19)	0.0493 (7)	
H23	0.3224	0.4681	−0.0301	0.059*	
C24	0.2981 (4)	0.2818 (3)	0.0286 (2)	0.0550 (7)	
H24	0.3710	0.2487	−0.0095	0.066*	
C25	0.2200 (4)	0.1994 (3)	0.0975 (2)	0.0572 (8)	
H25	0.2394	0.1106	0.1059	0.069*	
C26	0.1127 (3)	0.2490 (3)	0.1541 (2)	0.0497 (7)	
H26	0.0611	0.1927	0.2011	0.060*	
C27	0.4248 (6)	0.1793 (8)	0.3161 (4)	0.130 (2)	
H27A	0.3189	0.2096	0.3335	0.156*	
H27B	0.4875	0.1658	0.3663	0.156*	
C28	0.4742 (5)	0.2807 (4)	0.2334 (4)	0.0969 (16)	
H28A	0.4497	0.3640	0.2436	0.116*	
H28B	0.4169	0.2891	0.1822	0.116*	
Cl1	0.4405 (2)	0.03136 (15)	0.29818 (12)	0.1337 (6)	
Cl2	0.67477 (15)	0.24467 (12)	0.20429 (9)	0.0987 (4)	
N1	0.2825 (2)	0.6424 (2)	0.24224 (15)	0.0421 (5)	
N2	0.1009 (2)	0.5506 (2)	0.36207 (14)	0.0387 (5)	
N3	0.1477 (2)	0.6518 (2)	0.12931 (13)	0.0373 (5)	
N4	0.0168 (2)	0.5040 (2)	0.25176 (14)	0.0392 (5)	
O1	0.4116 (2)	0.6274 (2)	0.10852 (13)	0.0527 (5)	
O2	0.0510 (2)	0.34424 (19)	0.39298 (13)	0.0516 (5)	
O3	0.1344 (3)	0.8818 (2)	0.2469 (2)	0.0853 (8)	
O4	−0.0449 (3)	0.7988 (2)	0.3460 (2)	0.0856 (8)	
O5	−0.2343 (3)	0.7010 (3)	0.2211 (2)	0.0865 (9)	
O6	−0.1047 (2)	0.84312 (19)	0.12437 (15)	0.0582 (6)	
C14'	−0.178 (2)	0.9307 (14)	0.4359 (10)	0.083 (6)	0.298 (14)
H14D	−0.2692	0.9053	0.4272	0.125*	0.298 (14)
H14E	−0.2036	1.0188	0.4364	0.125*	0.298 (14)
H14F	−0.1368	0.8732	0.4933	0.125*	0.298 (14)
C13'	−0.062 (3)	0.9224 (17)	0.3593 (19)	0.109 (9)	0.298 (14)
H13C	−0.0937	0.9929	0.3033	0.131*	0.298 (14)
H13D	0.0366	0.9318	0.3737	0.131*	0.298 (14)

### Atomic displacement parameters (Å^2^)

**Table:**

	*U*^11^	*U*^22^	*U*^33^	*U*^12^	*U*^13^	*U*^23^
C1	0.0371 (13)	0.0563 (16)	0.0361 (13)	−0.0142 (11)	−0.0003 (10)	−0.0176 (12)
C2	0.0476 (16)	0.0627 (18)	0.0445 (15)	−0.0184 (13)	−0.0048 (12)	−0.0086 (13)
C3	0.0564 (19)	0.0537 (17)	0.0596 (18)	−0.0080 (14)	−0.0156 (15)	−0.0096 (14)
C4	0.0385 (15)	0.0658 (19)	0.068 (2)	−0.0054 (13)	−0.0085 (14)	−0.0292 (16)
C5	0.0338 (14)	0.0666 (18)	0.0494 (16)	−0.0145 (12)	0.0019 (12)	−0.0218 (14)
C6	0.0354 (13)	0.0540 (15)	0.0411 (14)	−0.0148 (11)	−0.0013 (11)	−0.0187 (12)
C7	0.0353 (14)	0.0592 (17)	0.0485 (15)	−0.0192 (12)	−0.0022 (11)	−0.0118 (13)
C8	0.0372 (14)	0.0653 (17)	0.0349 (13)	−0.0139 (12)	0.0024 (11)	−0.0176 (12)
C9	0.0352 (13)	0.0367 (13)	0.0410 (14)	−0.0095 (10)	0.0016 (11)	−0.0049 (10)
C10	0.0270 (12)	0.0450 (14)	0.0365 (13)	−0.0094 (10)	0.0044 (10)	−0.0105 (11)
C11	0.0305 (12)	0.0421 (13)	0.0402 (13)	−0.0094 (10)	0.0005 (10)	−0.0123 (11)
C12	0.0506 (17)	0.0498 (16)	0.0552 (17)	−0.0114 (13)	−0.0061 (14)	−0.0194 (14)
C14	0.196 (11)	0.081 (5)	0.103 (6)	0.005 (6)	−0.015 (6)	−0.044 (4)
C13	0.089 (5)	0.063 (4)	0.115 (5)	0.019 (3)	0.020 (4)	−0.036 (3)
C15	0.0323 (12)	0.0401 (13)	0.0359 (13)	−0.0097 (10)	0.0002 (10)	−0.0094 (10)
C16	0.0347 (14)	0.0491 (15)	0.0466 (15)	−0.0094 (11)	−0.0008 (11)	−0.0125 (12)
C17	0.0547 (19)	0.0559 (19)	0.076 (2)	0.0096 (15)	−0.0052 (16)	−0.0134 (16)
C18	0.099 (3)	0.054 (2)	0.103 (3)	0.013 (2)	0.000 (3)	0.000 (2)
C19	0.0405 (14)	0.0486 (15)	0.0423 (14)	−0.0183 (11)	0.0012 (11)	−0.0136 (12)
C20	0.0441 (14)	0.0478 (15)	0.0330 (13)	−0.0109 (11)	−0.0008 (11)	−0.0085 (11)
C21	0.0398 (14)	0.0502 (15)	0.0383 (13)	−0.0132 (11)	−0.0062 (11)	−0.0140 (11)
C22	0.0378 (13)	0.0494 (15)	0.0353 (13)	−0.0096 (11)	−0.0032 (10)	−0.0140 (11)
C23	0.0454 (15)	0.0612 (18)	0.0432 (15)	−0.0137 (13)	0.0013 (12)	−0.0189 (13)
C24	0.0495 (17)	0.0634 (19)	0.0596 (18)	−0.0066 (14)	0.0005 (14)	−0.0337 (15)
C25	0.0621 (19)	0.0514 (17)	0.0642 (19)	−0.0075 (14)	−0.0086 (15)	−0.0272 (15)
C26	0.0543 (17)	0.0487 (16)	0.0494 (16)	−0.0181 (13)	−0.0049 (13)	−0.0146 (13)
C27	0.073 (3)	0.229 (8)	0.118 (4)	−0.017 (4)	0.000 (3)	−0.105 (5)
C28	0.089 (3)	0.066 (2)	0.145 (4)	0.007 (2)	−0.055 (3)	−0.044 (3)
Cl1	0.1401 (13)	0.1025 (10)	0.1312 (12)	−0.0443 (9)	−0.0254 (10)	0.0126 (8)
Cl2	0.1068 (9)	0.0891 (8)	0.1020 (8)	−0.0310 (6)	0.0009 (7)	−0.0292 (6)
N1	0.0317 (11)	0.0554 (13)	0.0394 (12)	−0.0153 (9)	0.0016 (9)	−0.0134 (10)
N2	0.0312 (11)	0.0488 (12)	0.0351 (11)	−0.0116 (9)	0.0020 (8)	−0.0119 (9)
N3	0.0331 (11)	0.0408 (11)	0.0344 (11)	−0.0087 (8)	0.0027 (8)	−0.0090 (9)
N4	0.0401 (11)	0.0413 (11)	0.0369 (11)	−0.0146 (9)	0.0001 (9)	−0.0107 (9)
O1	0.0362 (10)	0.0659 (13)	0.0494 (11)	−0.0124 (9)	0.0105 (9)	−0.0143 (9)
O2	0.0540 (12)	0.0500 (11)	0.0436 (11)	−0.0175 (9)	−0.0005 (9)	−0.0034 (9)
O3	0.106 (2)	0.0513 (14)	0.101 (2)	−0.0313 (14)	0.0131 (17)	−0.0258 (14)
O4	0.0864 (18)	0.0585 (14)	0.101 (2)	−0.0014 (13)	0.0328 (16)	−0.0346 (14)
O5	0.0343 (12)	0.0731 (16)	0.119 (2)	−0.0072 (10)	0.0087 (13)	0.0011 (15)
O6	0.0463 (11)	0.0451 (11)	0.0637 (13)	0.0029 (9)	0.0031 (9)	−0.0041 (10)
C14'	0.103 (11)	0.060 (7)	0.082 (9)	0.005 (7)	−0.001 (8)	−0.031 (6)
C13'	0.102 (12)	0.102 (11)	0.117 (12)	−0.003 (8)	0.006 (9)	−0.044 (8)

### Geometric parameters (Å, °)

**Table:**

C1—C2	1.385 (4)	C16—O6	1.300 (3)
C1—C6	1.401 (4)	C17—C18	1.460 (5)
C1—C8	1.518 (4)	C17—O6	1.465 (4)
C2—C3	1.388 (4)	C17—H17A	0.9700
C2—H2	0.9300	C17—H17B	0.9700
C3—C4	1.377 (5)	C18—H18A	0.9600
C3—H3	0.9300	C18—H18B	0.9600
C4—C5	1.377 (4)	C18—H18C	0.9600
C4—H4	0.9300	C19—N4	1.453 (3)
C5—C6	1.391 (4)	C19—C21	1.520 (4)
C5—H5	0.9300	C19—H19A	0.9700
C6—C7	1.511 (4)	C19—H19B	0.9700
C7—N1	1.459 (3)	C20—N3	1.466 (3)
C7—H7A	0.9700	C20—C22	1.515 (4)
C7—H7B	0.9700	C20—H20A	0.9700
C8—N2	1.464 (3)	C20—H20B	0.9700
C8—H8A	0.9700	C21—C26	1.387 (4)
C8—H8B	0.9700	C21—C22	1.403 (4)
C9—O1	1.208 (3)	C22—C23	1.389 (4)
C9—N1	1.362 (3)	C23—C24	1.376 (4)
C9—N3	1.386 (3)	C23—H23	0.9300
C10—O2	1.206 (3)	C24—C25	1.375 (5)
C10—N4	1.367 (3)	C24—H24	0.9300
C10—N2	1.389 (3)	C25—C26	1.378 (4)
C11—N2	1.439 (3)	C25—H25	0.9300
C11—N1	1.441 (3)	C26—H26	0.9300
C11—C12	1.543 (4)	C27—C28	1.480 (8)
C11—C15	1.573 (3)	C27—Cl1	1.742 (7)
C12—O3	1.186 (4)	C27—H27A	0.9700
C12—O4	1.292 (4)	C27—H27B	0.9700
C14—C13	1.496 (8)	C28—Cl2	1.784 (5)
C14—H14A	0.9600	C28—H28A	0.9700
C14—H14B	0.9600	C28—H28B	0.9700
C14—H14C	0.9600	O4—C13'	1.438 (9)
C13—O4	1.455 (5)	C14'—C13'	1.500 (9)
C13—H13A	0.9700	C14'—H14D	0.9600
C13—H13B	0.9700	C14'—H14E	0.9600
C15—N3	1.435 (3)	C14'—H14F	0.9600
C15—N4	1.440 (3)	C13'—H13C	0.9700
C15—C16	1.547 (4)	C13'—H13D	0.9700
C16—O5	1.175 (3)		
			
C2—C1—C6	118.6 (3)	H18B—C18—H18C	109.5
C2—C1—C8	118.8 (2)	N4—C19—C21	113.5 (2)
C6—C1—C8	122.5 (2)	N4—C19—H19A	108.9
C1—C2—C3	121.4 (3)	C21—C19—H19A	108.9
C1—C2—H2	119.3	N4—C19—H19B	108.9
C3—C2—H2	119.3	C21—C19—H19B	108.9
C4—C3—C2	119.8 (3)	H19A—C19—H19B	107.7
C4—C3—H3	120.1	N3—C20—C22	115.3 (2)
C2—C3—H3	120.1	N3—C20—H20A	108.4
C3—C4—C5	119.3 (3)	C22—C20—H20A	108.4
C3—C4—H4	120.3	N3—C20—H20B	108.4
C5—C4—H4	120.3	C22—C20—H20B	108.4
C4—C5—C6	121.6 (3)	H20A—C20—H20B	107.5
C4—C5—H5	119.2	C26—C21—C22	119.2 (3)
C6—C5—H5	119.2	C26—C21—C19	119.6 (2)
C5—C6—C1	119.2 (3)	C22—C21—C19	121.3 (2)
C5—C6—C7	118.8 (2)	C23—C22—C21	118.6 (3)
C1—C6—C7	122.0 (2)	C23—C22—C20	119.3 (2)
N1—C7—C6	112.8 (2)	C21—C22—C20	122.1 (2)
N1—C7—H7A	109.0	C24—C23—C22	121.4 (3)
C6—C7—H7A	109.0	C24—C23—H23	119.3
N1—C7—H7B	109.0	C22—C23—H23	119.3
C6—C7—H7B	109.0	C25—C24—C23	119.9 (3)
H7A—C7—H7B	107.8	C25—C24—H24	120.0
N2—C8—C1	115.4 (2)	C23—C24—H24	120.0
N2—C8—H8A	108.4	C24—C25—C26	119.7 (3)
C1—C8—H8A	108.4	C24—C25—H25	120.1
N2—C8—H8B	108.4	C26—C25—H25	120.1
C1—C8—H8B	108.4	C25—C26—C21	121.2 (3)
H8A—C8—H8B	107.5	C25—C26—H26	119.4
O1—C9—N1	126.6 (2)	C21—C26—H26	119.4
O1—C9—N3	125.6 (2)	C28—C27—Cl1	112.0 (4)
N1—C9—N3	107.8 (2)	C28—C27—H27A	109.2
O2—C10—N4	126.3 (2)	Cl1—C27—H27A	109.2
O2—C10—N2	126.0 (2)	C28—C27—H27B	109.2
N4—C10—N2	107.6 (2)	Cl1—C27—H27B	109.2
N2—C11—N1	113.7 (2)	H27A—C27—H27B	107.9
N2—C11—C12	113.9 (2)	C27—C28—Cl2	112.9 (3)
N1—C11—C12	110.0 (2)	C27—C28—H28A	109.0
N2—C11—C15	103.24 (19)	Cl2—C28—H28A	109.0
N1—C11—C15	101.53 (19)	C27—C28—H28B	109.0
C12—C11—C15	113.7 (2)	Cl2—C28—H28B	109.0
O3—C12—O4	125.4 (3)	H28A—C28—H28B	107.8
O3—C12—C11	122.0 (3)	C9—N1—C11	113.3 (2)
O4—C12—C11	112.6 (2)	C9—N1—C7	124.3 (2)
O4—C13—C14	105.1 (6)	C11—N1—C7	122.2 (2)
O4—C13—H13A	110.7	C10—N2—C11	110.7 (2)
C14—C13—H13A	110.7	C10—N2—C8	120.4 (2)
O4—C13—H13B	110.7	C11—N2—C8	120.6 (2)
C14—C13—H13B	110.7	C9—N3—C15	111.0 (2)
H13A—C13—H13B	108.8	C9—N3—C20	120.1 (2)
N3—C15—N4	114.1 (2)	C15—N3—C20	120.6 (2)
N3—C15—C16	113.7 (2)	C10—N4—C15	113.5 (2)
N4—C15—C16	109.9 (2)	C10—N4—C19	123.9 (2)
N3—C15—C11	103.08 (18)	C15—N4—C19	122.6 (2)
N4—C15—C11	101.56 (19)	C12—O4—C13'	105.1 (7)
C16—C15—C11	113.7 (2)	C12—O4—C13	119.7 (5)
O5—C16—O6	125.1 (3)	C13'—O4—C13	23.8 (11)
O5—C16—C15	122.6 (3)	C16—O6—C17	115.7 (2)
O6—C16—C15	112.2 (2)	C13'—C14'—H14D	109.5
C18—C17—O6	107.9 (3)	C13'—C14'—H14E	109.5
C18—C17—H17A	110.1	H14D—C14'—H14E	109.5
O6—C17—H17A	110.1	C13'—C14'—H14F	109.5
C18—C17—H17B	110.1	H14D—C14'—H14F	109.5
O6—C17—H17B	110.1	H14E—C14'—H14F	109.5
H17A—C17—H17B	108.4	O4—C13'—C14'	110.2 (11)
C17—C18—H18A	109.5	O4—C13'—H13C	109.6
C17—C18—H18B	109.5	C14'—C13'—H13C	109.6
H18A—C18—H18B	109.5	O4—C13'—H13D	109.6
C17—C18—H18C	109.5	C14'—C13'—H13D	109.6
H18A—C18—H18C	109.5	H13C—C13'—H13D	108.1
			
C6—C1—C2—C3	−0.6 (4)	N2—C11—N1—C9	120.0 (2)
C8—C1—C2—C3	−177.7 (3)	C12—C11—N1—C9	−110.9 (2)
C1—C2—C3—C4	0.7 (5)	C15—C11—N1—C9	9.8 (3)
C2—C3—C4—C5	−0.4 (5)	N2—C11—N1—C7	−63.1 (3)
C3—C4—C5—C6	0.1 (5)	C12—C11—N1—C7	66.1 (3)
C4—C5—C6—C1	0.0 (4)	C15—C11—N1—C7	−173.2 (2)
C4—C5—C6—C7	179.3 (3)	C6—C7—N1—C9	−105.6 (3)
C2—C1—C6—C5	0.3 (4)	C6—C7—N1—C11	77.8 (3)
C8—C1—C6—C5	177.2 (2)	O2—C10—N2—C11	167.3 (2)
C2—C1—C6—C7	−179.0 (3)	N4—C10—N2—C11	−14.3 (3)
C8—C1—C6—C7	−2.0 (4)	O2—C10—N2—C8	18.7 (4)
C5—C6—C7—N1	125.7 (3)	N4—C10—N2—C8	−162.9 (2)
C1—C6—C7—N1	−55.0 (4)	N1—C11—N2—C10	−90.4 (2)
C2—C1—C8—N2	−125.1 (3)	C12—C11—N2—C10	142.5 (2)
C6—C1—C8—N2	57.9 (4)	C15—C11—N2—C10	18.7 (2)
N2—C11—C12—O3	150.2 (3)	N1—C11—N2—C8	58.1 (3)
N1—C11—C12—O3	21.2 (4)	C12—C11—N2—C8	−69.0 (3)
C15—C11—C12—O3	−91.9 (3)	C15—C11—N2—C8	167.2 (2)
N2—C11—C12—O4	−32.4 (3)	C1—C8—N2—C10	72.8 (3)
N1—C11—C12—O4	−161.4 (3)	C1—C8—N2—C11	−72.7 (3)
C15—C11—C12—O4	85.6 (3)	O1—C9—N3—C15	168.2 (2)
N2—C11—C15—N3	−134.01 (19)	N1—C9—N3—C15	−12.5 (3)
N1—C11—C15—N3	−16.0 (2)	O1—C9—N3—C20	19.6 (4)
C12—C11—C15—N3	102.1 (2)	N1—C9—N3—C20	−161.1 (2)
N2—C11—C15—N4	−15.6 (2)	N4—C15—N3—C9	−91.4 (2)
N1—C11—C15—N4	102.4 (2)	C16—C15—N3—C9	141.5 (2)
C12—C11—C15—N4	−139.5 (2)	C11—C15—N3—C9	17.9 (3)
N2—C11—C15—C16	102.4 (2)	N4—C15—N3—C20	57.1 (3)
N1—C11—C15—C16	−139.6 (2)	C16—C15—N3—C20	−70.1 (3)
C12—C11—C15—C16	−21.5 (3)	C11—C15—N3—C20	166.3 (2)
N3—C15—C16—O5	147.3 (3)	C22—C20—N3—C9	71.6 (3)
N4—C15—C16—O5	18.0 (4)	C22—C20—N3—C15	−74.0 (3)
C11—C15—C16—O5	−95.1 (3)	O2—C10—N4—C15	−178.7 (2)
N3—C15—C16—O6	−34.2 (3)	N2—C10—N4—C15	2.9 (3)
N4—C15—C16—O6	−163.5 (2)	O2—C10—N4—C19	0.3 (4)
C11—C15—C16—O6	83.4 (3)	N2—C10—N4—C19	−178.1 (2)
N4—C19—C21—C26	124.3 (3)	N3—C15—N4—C10	118.4 (2)
N4—C19—C21—C22	−56.0 (3)	C16—C15—N4—C10	−112.5 (2)
C26—C21—C22—C23	−0.7 (4)	C11—C15—N4—C10	8.3 (3)
C19—C21—C22—C23	179.7 (2)	N3—C15—N4—C19	−60.6 (3)
C26—C21—C22—C20	178.0 (2)	C16—C15—N4—C19	68.5 (3)
C19—C21—C22—C20	−1.6 (4)	C11—C15—N4—C19	−170.8 (2)
N3—C20—C22—C23	−122.5 (3)	C21—C19—N4—C10	−102.0 (3)
N3—C20—C22—C21	58.8 (3)	C21—C19—N4—C15	76.9 (3)
C21—C22—C23—C24	1.2 (4)	O3—C12—O4—C13'	−12.5 (15)
C20—C22—C23—C24	−177.6 (3)	C11—C12—O4—C13'	170.2 (14)
C22—C23—C24—C25	−0.7 (4)	O3—C12—O4—C13	8.0 (7)
C23—C24—C25—C26	−0.4 (5)	C11—C12—O4—C13	−169.3 (5)
C24—C25—C26—C21	0.8 (4)	C14—C13—O4—C12	−88.2 (9)
C22—C21—C26—C25	−0.3 (4)	C14—C13—O4—C13'	−32 (3)
C19—C21—C26—C25	179.3 (3)	O5—C16—O6—C17	4.0 (5)
Cl1—C27—C28—Cl2	−66.8 (4)	C15—C16—O6—C17	−174.5 (3)
O1—C9—N1—C11	−180.0 (2)	C18—C17—O6—C16	−177.6 (3)
N3—C9—N1—C11	0.7 (3)	C12—O4—C13'—C14'	−170.1 (18)
O1—C9—N1—C7	3.2 (4)	C13—O4—C13'—C14'	58.7 (18)
N3—C9—N1—C7	−176.2 (2)		

### Hydrogen-bond geometry (Å, °)

**Table:**

*D*—H···*A*	*D*—H	H···*A*	*D*···*A*	*D*—H···*A*
C28—H28B···Cg2	0.97	2.55	3.476 (3)	160
C5—H5···O5^i^	0.93	2.59	3.393 (4)	145
C7—H7B···O5^i^	0.97	2.50	3.359 (3)	148
C14—H14A···O2^ii^	0.96	2.57	3.514 (9)	168
C14'—H14F···O2^ii^	0.96	2.47	3.351 (6)	153

Symmetry codes: (i) *x*+1, *y*, *z*; (ii) −*x*, −*y*+1, −*z*+1.
